# Comments on Sabariego *et al*. Measuring Disability: Comparing the Impact of Two Data Collection Approaches on Disability Rates. *Int. J. Environ. Res. Public Health*, 2015, *12*, 10329–10351

**DOI:** 10.3390/ijerph13010065

**Published:** 2015-12-22

**Authors:** Jennifer H. Madans, Daniel Mont, Mitchell Loeb

**Affiliations:** 1 National Center for Health Statistics, 3311 Toledo Rd., Hyattsville, MD 20782, USA; gze1@cdc.gov; 2 Leonard Cheshire Disability and Development Centre, Department of Epidemiology and Public Health, University College London, 1-19 Torrington Place, London WC1E 6BT, UK; danielmont01@gmail.com

In the article, Measuring Disability: Comparing the Impact of Two Data Collection Approaches on Disability Rates, in Volume 12 of the Journal *International Journal of Environmental Research and Public Health*, Carla Sabariego *et al.* [1] raise several issues regarding the use of the short set of questions developed by the Washington Group on Disability Statistics (WG) as compared with the approach to disability measurement proposed through the Model Disability Survey (MDS). We address these below. We focus on broad conceptual and methodological issues rather than our concerns with how the WG questions are used in the MDS (e.g., specific wording and placement) or on the quality of the MDS questionnaire itself; however, it should be noted that these issues affect the validity of the results reported in the Sabariego article. 

The authors are concerned about using screeners in disability surveys. The purpose of screeners is to select a population of interest who will then be asked additional questions that would apply only to them. Screening questions are designed to maximize the chances that the population of interest will be captured even if some of those not of interest (false positives) are also identified as these cases can be eliminated from the population of interest based on the additional information collected. The issue is not whether a screener should be used, but whether the screener identifies the correct population.

In accordance with the framework proposed by the International Classification of Functioning, Disability and Health (ICF), the WG short set was designed to identify a population which, due to difficulty functioning in core domains, is at risk of restricted participation in a non-accommodating environment. The questions are intended to be used in conjunction with other information collected on national censuses and surveys, primarily to disaggregate outcomes of interest (e.g., employment, education) by disability status. The WG short set of six questions allows for the generation of multiple disability identifiers (levels of mild, moderate or severe difficulty based on six functional domains and four response options) that reflect the continuum of disability. Those with a disability can be compared to those without a disability on outcomes of interest such as employment, education, income, and use of services. Comparisons can also be made according to the extent of the disability (severity level). As noted above, the issue is not whether the WG questions work well as screeners or whether the MDS, a very long complicated survey, is a better data collection tool. Rather, the issue should be whether the appropriate population is identified for the analytic objective. The paper argues that the two approaches capture different populations and that one is better than the other. While the populations as defined in the analysis may be different, there is no evidence that the MDS population is more correct.

While other cut points can be used to identify lesser risk, when disability status is defined as a dichotomy, the WG has recommended cut points that identify a population that can be described as having moderate to high risk of not being able to participate in society (defined as having at least a lot of difficulty in at least one of the six WG domains). The selection of a cut point is a critical decision, but it should be kept in mind that the cut point can vary for different purposes. As long as the questions are the same across surveys, when different cut points are used, results from different analyses can be compared. It is known that the more heterogeneous the population defined as having a disability, the more similar that population will be to those without a disability so identifying a larger population is not an indicator that it is the right population. It is also interesting that given the authors interest in those with mild disability, they did not identify the population of interest as those with “some difficulty” in at least one of the domains covered. 

Both the MDS and the WG questions can be used to create a range of disability statuses by severity. The WG focuses on limitations in core functional domains so that comparisons in participation can be made by disability status. The MDS asks many more questions including both those addressing basic functioning as well as complex participation and then uses an IRT method to create a mathematically smoother measure of disability severity. Leaving aside whether the assumptions underlying the IRT model are met and whether the underlying dimension is “disability”, developing the distribution is independent of the notion of where a cut point should be drawn to differentiate people with and without disabilities. The authors recognize the importance of this cut point for disaggregating important development indicators by disability, but do not offer a clear conceptualization of how that cut point is determined. An advantage of the WG questions is that cut point is conceptually simple, clear, and easily constructed. To ask national statistical offices around the world to employ the MDS measure requires more resources and technical capacity but without a clear advantage of an underlying notion of how to identify a population(s) with disability. Besides, it is unclear what the advantage is of using a smoother measure if the primary purpose is to divide the population into discrete sub-populations. In a sense, it becomes more arbitrary where the cut point is placed because of the similarity of people on both sides of the cut point. The WG approach of a lot of difficulty versus no or some difficulty is clearer—though obviously no approach eliminates false positives or negatives.

Monitoring requires measurement at multiple time periods. Cost and burden on data collection systems limits the frequency and amount of information that can be collected. Monitoring is best supported when the information is collected through ongoing, institutionalized data systems. Short sets of questions can be incorporated into these systems so that the information for monitoring is available at multiple points, and disability measures across different data tools are consistent. Data collections like the MDS are costly, difficult to administer and require advanced analytic and data interpretation skills and are, therefore, unlikely to be adopted by countries and done multiple times. As a result, they are not good tools for monitoring the requirements of the UN Convention on the Rights of Person with Disabilities or the Post 2015 Sustainable Development Goals. In depth surveys can provide information to understand the mechanisms that are reflected in the indicators that are monitored on a regular basis. Conversely, short sets of questions are better suited for ongoing data collections to monitor the impact of policy changes. The WG question sets were developed to monitor change in the status of persons with disabilities. The paper by Carla Sabariego *et al*. [1] does not present any evidence that would question their ability to do so. Work is underway to evaluate what questions should be added to the short set so that they can better be used as screeners for survey designs that require screeners. The appropriateness of their use as a screener will depend on whether they capture all persons intended for follow-up questions.
